# Supplementary material for: Loss of α-actinin-3 during human evolution provides superior cold resilience and muscle heat generation
Source: Am J Hum Genet. 2021 Feb 17;108(3):446–57. doi: 10.1016/j.ajhg.2021.01.013 (PMC8008486; doi:10.1016/j.ajhg.2021.01.013)
Supplement: Document S1. Figures S1–S4 and Tables S1 and S2 [file mmc1.pdf]

The American Journal of Human Genetics, Volume *108*

Supplemental data

Loss of α-actinin-3 during human evolution provides superior cold resilience and muscle heat generation

Victoria L. Wyckelsma, Tomas Venckunas, Peter J. Houweling, Maja Schlittler, Volker M. Lauschke, Chrystal F. Tiong, Harrison D. Wood, Niklas Ivarsson, Henrikas Paulauskas, Nerijus Eimantas, Daniel C. Andersson, Kathryn N. North, Marius Brazaitis, and Håkan Westerblad

Supplemental Data

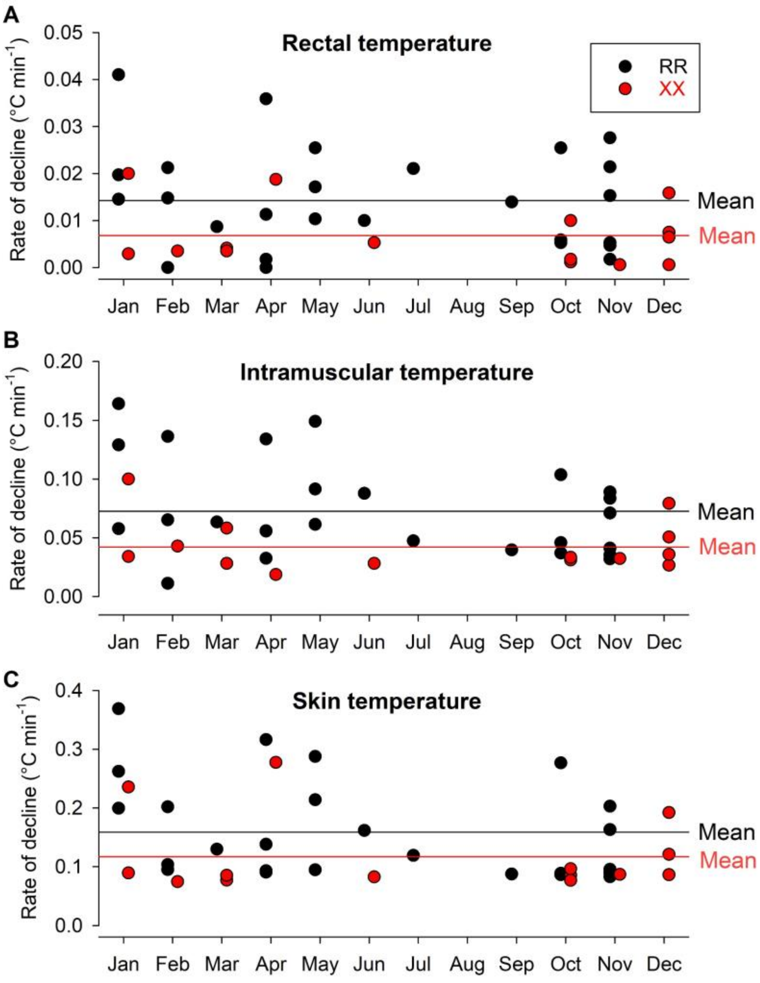

Figure S1 Rates of temperature decline during cold-water immersion did not show any notable dependency on when during the year experiments were performed.

The rates of rectal (**A**), intramuscular (**B**), and skin (**C**) temperature decline during cold-water immersion plotted against the month of the year when the experiment was performed. Plots show values for each RR (black circles) and XX (red circles) individual. Lines show the mean values for the two groups.

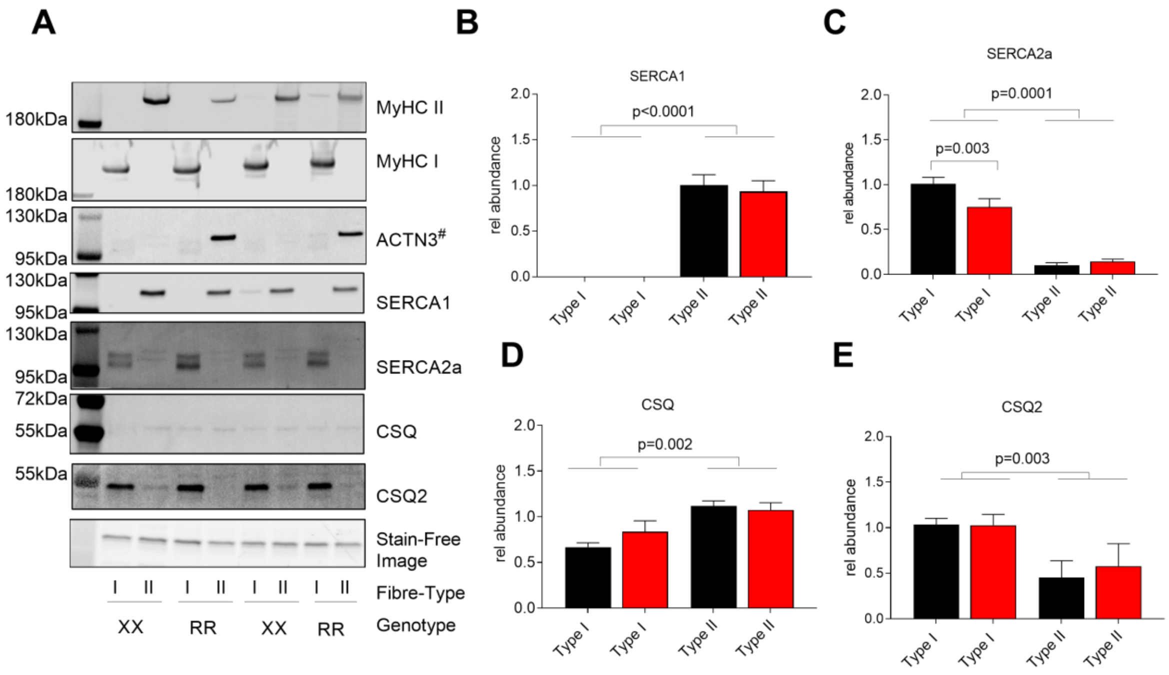

Figure S2 Fiber type-specific measurements of SR Ca^{2+}-ATPase and calsequestrin protein expression in muscle of RR and XX individuals.

Representative blots (**A**) and summary data of pooled single fiber expression of SERCA1 (**B**), SERCA2a (**C**), CSQ (**D**), and CSQ2 (**E**) in RR (black bars) and XX (red bars) individuals. Fibers were identified as either Type I (MyHC I) or Type II (MyHC II). α-Actinin-3 has a similar molecular weight as both SERCA1 and 2a, hence it was not possible to probe for α-actinin-3 on the same membrane as SERCA1 and 2 and an extra gel was run for α-actinin-3 (marked ACTN3#). Stain free images show the actin prior to transfer, which was used as a loading control. For each single fiber pool, proteins were normalized against their own calibration curve (~5-40 µg wet weight protein) and total protein and expressed relative to the mean of the RR fibers, which was set to 1; proteins mainly expressed in Type I fibers were expressed relative to the mean of the RR Type I fibers and vice versa for proteins mainly expressed in Type II fibers. Data are presented as mean ± SEM. Differences between RR and XX were determined by one-way ANOVA with Tukey´s *post hoc* test.

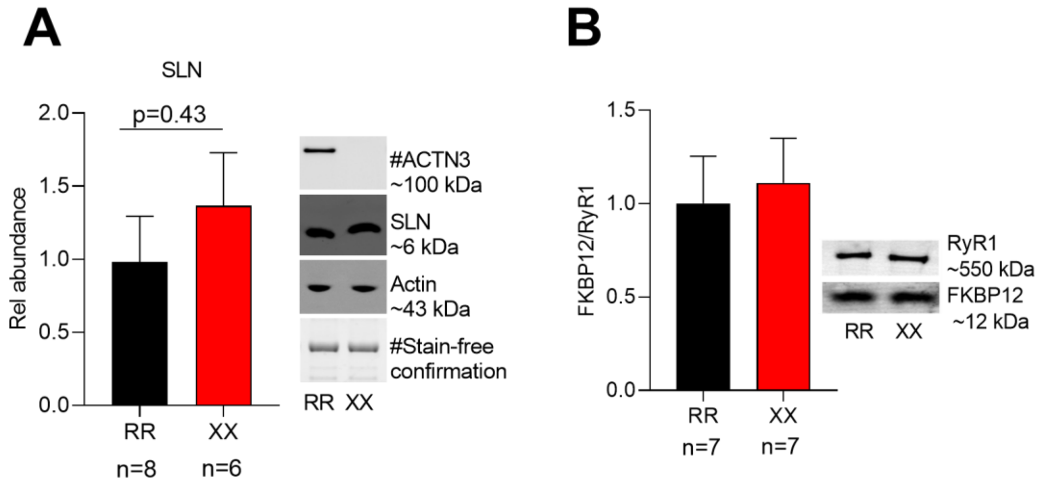

Figure S3 Measurements of sarcolipin protein expression and FKBP12 associated with the ryanodine receptor 1 in muscle of RR and XX individuals

Summary data (mean ± SEM) and representative whole muscle homogenate western blots of SLN (**A**) and FKBP12 associated with RyR1 (**B**). Band intensities in **A** were normalized to their respective actin stain-free loading controls; # in **A** indicates a separate gel from that of SLN (qualitatively similar results obtained with actin on same gel used as loading control). Data are expressed relative to the mean value of the RR group, which was set to 1.0. No statistical difference (*P* > 0.05) in SLN expression or FKBP12 associated with RyR1 between RR and XX individuals were observed with unpaired t-test.

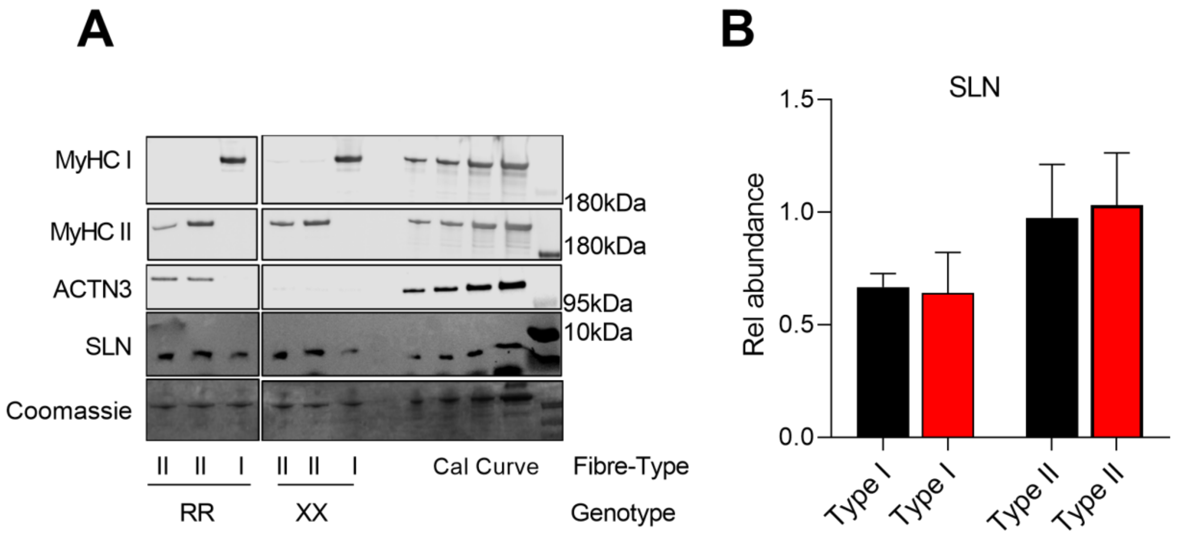

Figure S4 Fiber type-specific measurements of sarcolipin protein expression in muscle of RR and XX individuals.

(A) Representative blots for analysis of SLN protein expression in pooled single fibers. SLN was run on a 16.5% Tris Glycine Gel and Coomassie stained after transfer for loading control. The two MyHC isoforms and α-actinin-3 were run on two different gels, loaded identical to those described for SLN. Fibers are identified as either Type I (MyHC I), Type II (MyHC II). **B**) For each single fiber pool, SLN was normalized against its own calibration curve and protein content and expressed relative to the mean of the RR MyHC II, which was set to 1. Data are shown as mean ± SEM; RR, black bars; XX, red bars.

	ACTN3 genotype groups	
	**RR (n=27)**	**XX (n=15)**
Age, yrs	22 ± 1	28 ± 2*
Height, cm	181 ± 1	183 ± 2
Body weight, kg	77.2 ± 2.5	81.2 ± 3.5
Body surface area, m^2	1.96 ± 0.03	2.01 ± 0.05
Mean skinfold thickness, mm	12.1 ± 0.9	14.5 ± 1.3
Body mass index, $kg \cdot m^{-2}$	23.6 ± 0.6	24.3 ± 0.7
Body fat, %	15.7 ± 1.0	17.9 ± 1.1
Rectal T, °C	36.9 ± 0.1	36.8 ± 0.1
Calf muscle T, °C	35.6 ± 0.1	35.2 ± 0.2
Skin T, °C	32.1 ± 0.1	31.8 ± 0.2

Table S1 Physical characteristics and baseline temperatures of the individuals in the RR and XX groups.

Values are mean ± SEM. * $P < 0.05$ in unpaired t-test. Thus, RR subjects were slightly younger than XX subjects, whereas no statistically significant differences were observed for any of the other measured physical properties or baseline temperatures.

**Full name**	**Symbol**	**NCBI Reference Sequence**
DnaJ heat shock protein family (Hsp40) member C27	*Dnajc27*	NM_153082.4
Ganglioside-induced differentiation-associated-protein 2	*Gdap2*	NM_010269.3
Glutamic-oxaloacetic transaminase 1, soluble	*Got1*	NM_010324.2
Glycerol-3-phosphate dehydrogenase 1-like	*Gpd1l*	NM_175380.5
interleukin-1 receptor-associated kinase 2	*Irak2*	NM_001113553.1
Kelch domain containing 7A (	*Klhdc7a*	NM_173427.2
Peroxisome proliferative activated receptor, gamma, coactivator 1 alpha	*Ppargc1a*	NM_008904.2
Pleckstrin homology domain containing, family M (with RUN domain) member 2	*Plekhm2*	NM_001033150.2
Procollagen-proline, 2-oxoglutarate 4-dioxygenase (proline 4-hydroxylase), alpha 1 polypeptide	*P4ha1*	NM_001316370.1
Tectorin beta	*Tectb*	NM_009348.4

Table S2 Genes showing the largest response to acute cold exposure in mouse brown adipose tissue (BAT).

Genes identified in volcano plot of RNA-sequencing data in Figure 4D.
